# A Case of Acute Myelogenous Leukemia in Long‐Term Remission After Severe Infection Following Only One Course of Chemotherapy

**DOI:** 10.1155/crh/5567757

**Published:** 2026-05-12

**Authors:** Motoharu Shibusawa

**Affiliations:** ^1^ Department of Hematology, Sanaikai General Hospital, Saitama, Japan

## Abstract

Acute myelogenous leukemia (AML) is characterized by the uncontrolled growth of tumor cells in the bone marrow. Standard treatment includes induction remission therapy followed by several courses of consolidation chemotherapy. The 5‐year disease‐free survival rate is only 39%. A 61‐year‐old man with AML achieved remission after standard induction therapy but experienced severe myelosuppression and a serious infection, leaving him too exhausted for further chemotherapy. Remarkably, he remained in remission for over 5 years. This case suggests that both the initial chemotherapy and the immune response triggered by severe infection may have contributed to long‐term remission.

## 1. Introduction

Acute myelogenous leukemia (AML) is a disease caused by the proliferation of tumor cells in the bone marrow. Standard treatment for AML consists of induction remission therapy, followed by consolidation therapy, with a total of five to six courses of chemotherapy. According to a previous report, 77.5% of AML patients who undergo induction remission therapy with daunomycin and cytarabine achieve remission. However, even after four courses of consolidation therapy, the 5‐year disease‐free survival rate is only 39%. Thus, the majority of AML patients eventually relapse [1].

Reportedly, among patients with AML, remission can be achieved without chemotherapy. This remission is called spontaneous remission (SR). In a study analyzing 46 cases of SR in AML, 91.3% of the patients experienced a febrile episode prior to remission, most commonly due to pneumonia (54.5%) and bacteremia (24.2%). However, the median duration of remission with SR is as short as 5 months [2].

Currently, the mechanism of SR in AML is unclear. However, in previous reports, most patients developed infections, suggesting an association between SR and the development of infectious diseases. Furthermore, the median duration of remission is only 5 months.

We report the case of an AML patient who, after receiving only one course of chemotherapy, developed a severe infection and subsequently achieved long‐term remission without further chemotherapy. To the best of our knowledge, this is the first report documenting such a clinical course in AML, indicating a possible relationship between severe infection and long‐term remission.

## 2. Case presentation

**FIGURE 1:**
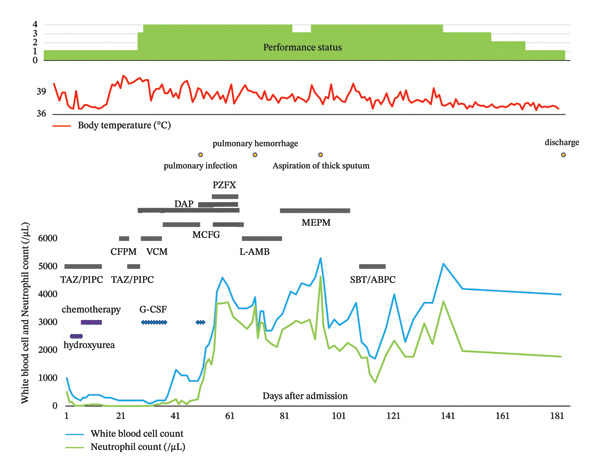
Clinical course after admission following chemotherapy, the patient developed a severe infection secondary to neutropenia. The patient’s performance status remained at 4. With the resolution of the infection and subsequent rehabilitation, the performance status gradually improved to 1.

A 61‐year‐old male with a history of pneumonia presented to our hospital. Seven days prior to admission, he experienced fever and watery diarrhea and consulted his previous physician. Laboratory tests revealed pancytopenia and elevated C‐reactive protein (CRP), raising suspicion of a hematologic disease. He was subsequently referred to our hospital for further evaluation and admitted. Figure 1 illustrates the clinical course following admission. On presentation, the patient had an Eastern Cooperative Oncology Group (ECOG) performance status of 1. His vital signs were body temperature, 40.2°C; blood pressure, 115/62 mmHg; heart rate, 95 bpm; and oxygen saturation (SpO_2_), 97%. Chest auscultation revealed no rales. The abdomen was flat and soft, and hepatosplenomegaly was not palpable. Initial laboratory findings were as follows: white blood cell count, 1000/μL (neutrophils 45%, eosinophils 1%, lymphocytes 48%, and atypical lymphocytes 1%); hemoglobin, 8.1 g/dL; platelet count, 2.3 × 10^4^/μL; lactate dehydrogenase, 1161 IU/L; CRP, 42.49 mg/dL; prothrombin time‐international normalized ratio (PT‐INR), 14.9 s; activated partial thromboplastin time (aPTT), 54.1 s; fibrinogen, 435 mg/dL; D‐dimer, 40.0 μg/mL; and fibrinogen degradation products, 111.8 μg/mL. A bone marrow examination revealed hypercellular marrow with increased blasts with a high nuclear‐to‐cytoplasmic (N/C) ratio, delicate nuclear chromatin, and pale cytoplasm. Some blasts had differentiated into monocyte‐like cells. The blasts were negative for peroxidase staining and positive for nonspecific esterase staining, comprising 90.8% of nucleated cells. Flow cytometry using CD45 gating demonstrated the following immunophenotype: HLA‐DR (+), CD33 (+), and CD56 (+). The patient was diagnosed with AML, French–American–British (FAB) classification M5. Chromosomal analysis using G‐banding showed a normal karyotype. Reverse transcription‐polymerase chain reaction detected 7.2 × 10^3^ copies of the KMT2A‐MLLT3 fusion gene.

The clinical course is demonstrated in Figure 1. On admission, empiric antibiotic therapy was initiated after collecting two blood cultures. The laboratory data also exhibited signs of disseminated intravascular coagulation, for which thrombomodulin was administered. On Day 3 postadmission, hydroxyurea (2000 mg) was started for cytoreduction. Induction chemotherapy with daunorubicin (50 mg/m^2^, 5 days) and cytarabine (100 mg/m^2^, 7 days) was initiated on Day 7. On Day 16, the patient developed a fever of 38.1°C. Blood cultures were obtained, and empiric antibiotics were started. On Day 18, *Corynebacterium striatum* was identified in the blood culture, and vancomycin therapy was started. A bone marrow examination on Day 22 showed hypoplastic marrow with a nucleated cell count of 0.7 × 10^4^/μL. Normal hematopoiesis was minimally observed. No leukemic blasts were identified. Granulocyte colony‐stimulating factor (G‐CSF) was initiated on Day 23. On Day 44, computed tomography (CT) revealed bilateral pulmonary consolidations, indicating the presence of pulmonary infection, and empiric antibiotic therapy was continued. However, Gram staining and culture of sputum samples did not reveal significant bacterial growth. He required intubation and ventilatory support. A bone marrow examination on Day 45 revealed hypoplastic marrow with evidence of hematopoiesis and no leukemic blasts. On Day 46, the patient was weaned off ventilator support. Following ICU discharge, he had an ECOG performance status of 4. Due to difficulty with oral intake, the patient depended on tube feeding. The patient became severely exhausted, and no further planned chemotherapy could be performed. Transthoracic echocardiography on Day 53 showed no evidence of infective endocarditis. On Day 58, bronchoscopy was performed, and cultures of bronchoalveolar lavage fluid were negative for significant pathogens. On Day 59, CT imaging revealed right upper lobe pulmonary infiltrates and a low‐density pelvic lesion suggestive of a hematoma. On Day 64, the patient produced a large volume of bloody sputum. A chest x‐ray on Day 65 showed diffuse bilateral pulmonary infiltrates, and pulmonary hemorrhage was diagnosed, presumed secondary to thrombocytopenia. The patient developed respiratory failure, necessitating intubation and mechanical ventilation. His respiratory status improved by Day 72, and he was extubated. However, on Day 87, the patient aspirated thick sputum, which necessitated intubation again and ventilatory support. Although primary mechanical ventilation was required, the patient eventually recovered and was successfully weaned off the ventilator.

Following continued rehabilitation, the patient gradually regained functional independence. On Day 183, he was discharged and transitioned to outpatient follow‐up. He has remained in remission for approximately 5 years since discharge.

## 3. Discussion

This case is notable in that the patient with AML achieved long‐term remission after receiving only a single course of chemotherapy.

An association between SR and infections has been reported [2]. Case reports have indicated this association, describing a patient with AML who experienced three separate episodes of infection, each followed by remission. In this case, the patient experienced three separate episodes of infection caused by different pathogens: nontuberculous acidosis, pulmonary aspergillosis, and Escherichia coli bacteremia. The patient might have had remission because of the disappearance of blasts from the peripheral blood, improvement of pancytopenia, and transfusion independence. Each of the three remissions was transient, and the duration of each of the three remissions was 3, 5, and 3 months [3]. This case report also suggests that the incidence of infection is closely related to the achievement of SR. However, remission due to infection was short‐lived, ranging from 3 to 5 months.

Our literature search did not identify any reported cases of AML developing after a severe infection following a single course of chemotherapy that resulted in long‐term remission. However, two cases of acute lymphoblastic leukemia (ALL) with long‐term remission after a short course of chemotherapy, similar to the present case, have been reported. Case 1 (46‐year‐old man) received dexamethasone and cyclophosphamide (1200 mg) as pretreatment and rituximab with a cumulative dose of 200 mg daunorubicin and 2 mg vincristine. After chemotherapy, he developed pulmonary mucormycosis and was treated with antifungal agents. He underwent a pneumonectomy. Case 2 (19‐year‐old man) was treated with (vincristine 1 mg, dexamethasone, daunorubicin 80 mg, mercaptopurine 12 months maintenance). After chemotherapy, the patient presented with elevated serum Aspergillus antigen and radiographic lung lesions indicative of pulmonary mycosis. He was treated with antifungal agents. In both cases, the infections were successfully treated, and complete remission of ALL was achieved. However, chemotherapy could not be continued. The remission durations were 34 months (Case 1) and 8 years (Case 2), respectively [4]. These two cases are similar to the present case in that the clinical course involved infections after shortened chemotherapy protocols, leading to discontinuation of chemotherapy; however, long‐term remission was achieved. The present case, nevertheless, differs from the aforementioned cases in terms of leukemia subtype: the present case is AML, whereas the two cases are ALL. The median reported remission duration associated with SR triggered by infection alone is approximately 5 months [2]. Compared with this, the remission durations observed in the two cases are notably longer. This suggests that the combination of a short course of chemotherapy and a severe infection may contribute to prolonged remission more effectively than infection alone.

In addition, some cases suggest a potential link between G‐CSF administration and remission in AML [5, 6]. Three cases of AML with hypoplastic bone marrow achieving remission after G‐CSF administration alone have been reported. In the first case, cytogenetic remission lasted over 9 months; in the second, 6 months; and in the third, remission was ongoing at 9 months posttreatment. Notably, all three patients did not experience concurrent infections [5]. The present case also involved G‐CSF administration, which may have contributed to the observed long‐term remission. It is noteworthy that all previously reported cases in which remission was achieved following the administration of G‐CSF involved AML with hypoplastic bone marrow. In contrast, the present case demonstrated hyperplastic bone marrow, thereby differing from previously documented cases. Although the underlying mechanism remains speculative, it is conceivable that G‐CSF–induced remission in AML occurs predominantly in cases characterized by hypoplastic marrow and a relatively low tumor burden. In the present case, the bone marrow was hyperplastic at initial presentation; however, G‐CSF was administered after chemotherapy, when the tumor burden had already decreased significantly. This clinical context may have contributed to achieving remission following G‐CSF administration.

The long‐term remission observed in the present case can be attributed to several factors. Initially, chemotherapy reduced the leukemic cell burden through its direct antitumor effects. It is hypothesized that the remaining minimal residual AML cells were subsequently eradicated by tumor‐specific immune responses, which were stimulated by cytokine production during a severe infection (6, 7). Additionally, G‐CSF administration may contribute to the long‐term remission.

## 4. Conclusion

The long‐term maintenance of remission observed in this case of AML may have been influenced not only by a single course of chemotherapy but also by the occurrence of a severe infectious disease.

**Nomenclature**
Performance status: Eastern Cooperative Oncology Group performance status

**Antibiotics**
TAZ/PIPC: Tazobactam/piperacillin
CFPM: Cefepime
VCM: Vancomycin
DAP: Daptomycin
MCFG: Micafungin
PZFX: Pazufloxacin
L‐AMB: Liposomal amphotericin B
MEPM: Meropenem

## Funding

No funding was received for this manuscript.

## Disclosure

The author has read and approved the final version of the manuscript. Motoharu Shibusawa had full access to all of the data in this study and takes complete responsibility for the integrity of the data and the accuracy of the data analysis.

## Consent

The patient allowed personal data processing, and informed consent was obtained from all individual participants included in the study.

## Conflicts of Interest

The author declares no conflicts of interest.

## Supporting Information

Additional supporting information can be found online in the Supporting Information section.

## Supporting information


**Supporting Information** This case report was prepared in accordance with the 2013 CARE guidelines. The CARE checklist is a consensus‐based reporting guideline designed to improve the completeness and transparency of published case reports. It comprises 13 checklist items covering: title, key words, abstract, introduction, patient information, clinical findings, timeline, diagnostic assessment, therapeutic interventions, follow‐up and outcomes, discussion, patient perspective, and informed consent.

## Data Availability

The data that support the findings of this study are available on request from the corresponding author. The data are not publicly available due to privacy or ethical restrictions.
